# Sural/Radial Amplitude Ratio: A Useful Tool to Diagnose Non‐Length‐Dependent Neuropathy

**DOI:** 10.1002/mus.70046

**Published:** 2025-10-09

**Authors:** Antoine Pegat, Antoine Gavoille, Florent Cluse, Martin Moussy, Philippe Petiot, Jean‐Philippe Camdessanché, Françoise Bouhour

**Affiliations:** ^1^ Hôpital Neurologique Pierre Wertheimer Service d’ENMG et de Pathologies Neuromusculaires, Centre de Référence des Maladies Neuromusculaires PACA‐Réunion‐Rhône Alpes, Hospices Civils de Lyon Bron France; ^2^ Hospices Civils de Lyon Service de Neurologie, Sclérose en Plaques, Pathologies de la Myéline et Neuro‐Inflammation Bron France; ^3^ Laboratoire de Biométrie et Biologie Évolutive UMR 5558 Université Lyon 1, CNRS Villeurbanne France; ^4^ Service de Biostatistique‐Bioinformatique Hospices Civils de Lyon Lyon France; ^5^ Service de Neurologie Centre Hospitalier Universitaire Saint‐Etienne France

**Keywords:** electroneuromyography, ENMG, non‐length dependent neuropathy, SRAR, sural/radial nerve amplitude ratio

## Abstract

**Introduction/Aims:**

Patients with non‐length‐dependent neuropathy (NLDN) exhibit reduced sensory nerve action potential (SNAP) amplitudes in both lower and upper limbs. This study aimed to determine a threshold for the sural/radial amplitude ratio (SRAR) suggestive of NLDN.

**Methods:**

This retrospective case–control study involved 60 patients with definite NLDN (sensory neuronopathy [SNN] or chronic inflammatory demyelinating polyradiculoneuropathy [CIDP]) and 30 patients with length‐dependent neuropathy (LDN). The diagnostic performance of SRAR was evaluated using the area under the curve (AUC) of the modeled receiver operating characteristic (ROC) curve. The presence of a length‐dependent electrodiagnostic (EDX) pattern, defined as a sural SNAP amplitude lower than the radial one, was evaluated in each group.

**Results:**

SRAR could be calculated in 90/164 (54.9%) of patients screened. Among patients with NLDN, the median SRAR was 0.74 (IQR 0.50–1.00) compared to 0.17 (IQR 0.12–0.23) in patients with LDN. The ROC curve analysis for NLDN versus LDN yielded an AUC of 0.94 (95% CI, 0.883–0.979). The SRAR threshold of 0.33 provided a sensitivity of 84.4% (95% CI, 77.8%–90.9%), specificity of 86.9% (95% CI, 79.7%–94%). The length‐dependent EDX pattern was observed in 100% (30/30) of LDN patients and 63% (38/60) of NLDN patients. Among these 38 patients with NLDN, SRAR exceeded 0.33 in 78.9% (30/38).

**Discussion:**

SRAR appears to be useful in the electrophysiological evaluation of neuropathies. In addition to usual diagnostic criteria, an SRAR > 0.33 may strongly suggest NLDN such as SNN or CIDP.

**Abbreviations**
Ago: Argonaute
AUC: area under the curve
CANVAS: cerebellar ataxia, neuropathy, vestibular areflexia syndrome
CI: confidence interval
CIDP: chronic inflammatory demyelinating polyradiculoneuropathy
EDX: electrodiagnostic studies
FGFR3: fibroblast growth factor receptor 3
IQR: interquartile range
LDN: length‐dependent neuropathies
NLDN: non‐length‐dependent neuropathy
NPV: negative predictive value
PPV: positive predictive value
RFC1: replication factor C subunit 1
ROC: receiver operative characteristic
SCA: spinocerebellar ataxia
SD: standard deviation
SNAP: sensory nerve action potential
SNN: sensory neuronopathies
SRAR: sural/radial nerve amplitude ratio

## 1 Introduction

The identification of a non‐length‐dependent neuropathy (NLDN), such as a sensory neuronopathy (SNN) or chronic inflammatory demyelinating polyradiculoneuropathy (CIDP), is critical for clinicians and neurophysiologists using electrodiagnostic (EDX) studies, clinical phenotype, and symptom evolution, since it provides important insights regarding potential etiologies, thus facilitating best management [1, 2].

SNN, a rare and heterogeneous group of diseases characterized by the selective primary degeneration of sensory neurons in the dorsal root ganglia, is classically diagnosed using Camdessanche criteria, which combine clinical and EDX data, with excellent diagnostic sensitivity and specificity especially in acquired SNN [3, 4, 5, 6]. In the most recent CIDP criteria, sensory nerve conduction criteria have been added; among them, the “sural sparing pattern” (corresponding to normal sural sensory nerve action potential [SNAP] amplitude with abnormal median or radial SNAP amplitude), observed in approximately 30% of cases [1, 7].

The sural/radial nerve amplitude ratio (SRAR) was proposed initially as a tool to assist in the diagnosis of length‐dependent neuropathy (LDN), based on the “dying‐back” pathophysiological mechanism, but it has also been used less commonly for the diagnosis of NLDN [8, 9, 10, 11, 12, 13, 14]. Patients with LDN typically exhibit decreased SNAP amplitudes in the lower limbs; the upper limbs may also be affected but to a lesser extent. This is in contrast to patients with NLDN who frequently demonstrate an early and severely reduced radial SNAP amplitude compared to lower limb SNAPs. The objective of the present study was to determine a threshold SRAR value suggestive of NLDN.

## 2 Methods

### 2.1 Study Design and Patient Selection

This retrospective case–control study was based on the medical records of patients referred between March 2021 and May 2024 to the neurophysiology department of the reference center for neuromuscular disorders at Lyon‐Bron, France. All consecutive patients with a diagnosis of SNN, CIDP, or LDN, who presented at least one abnormal value of SNAP for the radial and/or sural sensory nerve, were included until the inclusion of 30 patients with calculable SRAR for each type of neuropathy. The SRAR calculation required that the patients had undergone EDX studies demonstrating at least one recordable SNAP from both the sural and the radial sensory nerves. The SRAR was calculated by dividing the sural SNAP amplitude by the radial SNAP amplitude. When both sides were assessed, the lower SNAP value was used. When multiple EDX studies were available for a patient, only the first study was taken into account.

Then, the patients were divided into two groups. The NLDN group included patients with probable or possible SNN (based on Camdessanche criteria) [6] and CIDP diagnoses (CIDP, or possible CIDP, including typical and variants, excluding motor CIDP) based on EAN/PNS 2021 criteria [1]. SNN etiologies comprised inherited conditions (CANVAS/*RFC1*‐neuropathy [Cerebellar Ataxia, Neuropathy, Vestibular Areflexia Syndrome/Replication Factor C subunit 1] or Friedreich ataxia) and acquired causes [3, 4, 5, 15] (paraneoplastic, anti‐Hu or CV2 neuropathies, cisplatin‐induced neuropathy, dysimmune neuropathies [those associated with Sjögren syndrome (based on 2016 ACR/EULAR criteria) [16] or related to fibroblast growth factor receptor 3 antibodies (anti‐FGFR3) [17] or Argonaute antibodies (anti‐Ago)] [18], vitamin B12 deficiency [19], or idiopathic SNN). The LDN group included patients who exhibited clinically sensory or sensory‐motor neuropathy with a length‐dependent distribution and without meeting the Camdessanche criteria for SNN [6]. Etiologies of LDN included diabetes mellitus, vitamin deficiencies, toxic mechanisms, intensive care unit neuropathy, connective tissue diseases, hereditary transthyretin (*TTR*) amyloidosis, Charcot–Marie–Tooth type 2, or chronic idiopathic axonal polyneuropathy. In this group, patients were selected when they had at least one sural SNAP amplitude below the lower limit of normal value, based on our routine laboratory references.

All EDX studies were conducted by experienced electrophysiologists using Nicolet Viking EDX systems (Natus Medical Incorporated, Middleton, WI, USA). Standard methods with conventional surface electrodes were employed following current practice guidelines [20]. Sensory nerve recordings used antidromic techniques, with SNAP amplitudes measured peak‐to‐peak after supramaximal stimulation. No predetermined distance was used, based on our routine laboratory protocols. For the radial sensory nerve, the active electrode was placed at the base of the anatomical snuffbox and the reference electrode was placed 4 cm distally on the first dorsal interosseous space; the stimulation was performed on the distal–mid radius [21]. For the sural sensory nerve recording, the active electrode was placed posterior to the lateral malleolus and the reference electrode was placed 4 cm distally; the stimulation occurred on the posterior–lateral calf [22]. A length‐dependent EDX pattern was defined as a sural SNAP amplitude lower than the radial SNAP amplitude, irrespective of ratio, as previously described [23].

The study was approved by the institutional review board of the Hospices Civils de Lyon (HCL; approval number 24_197) and registered in the *Commission Nationale de l'Informatique et des Libertés* (CNIL; number HCL 24_5197).

### 2.2 Statistical Analyses

Continuous variables were expressed as median and interquartile range (IQR), and categorical variables by count and percentage. The correlation of SRAR with patient age was assessed using Pearson's correlation coefficient, with sex using Wilcoxon's test, and with the exact etiology in the SNN group (immune‐mediated, inherited, or other) using the Kruskal–Wallis test.

To determine the diagnostic performances of the SRAR in discriminating NLDN from LDN, its distribution was modeled using a parametric binormal function, after a logarithm transformation to improve its normality. The Area Under the Curve (AUC) of the SRAR was calculated based on the parametric Receiver Operative Characteristic (ROC) curve. To optimize the choice of the best threshold, a utility function was estimated, incorporating a pre‐test probability parameter (corresponding to the pre‐test probability of NLDN) and a risk cut‐off preference (which represents the probability of disease above which the physician will accept to initiate additional tests) [24]. In our neurophysiology department, the proportion of NLDN among all the EDX studies performed for polyneuropathy was 15% (data not shown), corresponding to the pre‐test probability. For the risk cut‐off preference, a panel of experts (A.P., F.C., M.M., F.B.) chose a value at 15%, which is equivalent to agreeing to wrongly perform additional tests in 5.7 patients with a LDN in order to avoid missing one diagnosis of NLDN (15% of patients with an additional assessment will really have a final diagnosis of SNN or CIDP). Of note, the Youden index, often used to determine an optimal threshold, is implicitly equivalent as assuming that false positives are as undesirable as false negatives (a risk cutoff preference of 50%) and that the prevalence of NLDN was 50% (a pre‐test probability of 50%). The sensitivity, specificity, positive predictive value (PPV), and negative predictive value (NPV) of the rounded optimal threshold (threshold for ease of use in practice) were calculated, assuming a 15% pre‐test probability of NLDN for the PPV and NPV.

As a sensitivity analysis, the optimal threshold according to different hypotheses of pre‐test probability and risk cut‐off preference was calculated. All confidence intervals (CI) were inferred using bootstrap on 2000 replicates with the percentile method. To assess the potential association between etiologies and SNN, the etiologies were grouped into three categories: immune‐mediated, inherited, and other. Statistical analyses were performed using R software (version 4.0.3) with the pROC package.

## 3 Results

### 3.1 Characteristics of Patients

**FIGURE 1:**
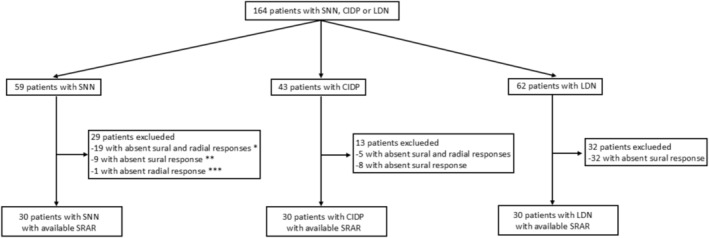
Flow chart of patients with available SRAR. CIDP, chronic inflammatory demyelinating polyradiculoneuropathy; LDN, length‐dependent neuropathy; SNN, sensory neuronopathy; SRAR, sural radial amplitude ratio. *: 10 CANVAS/RFC1‐neuropathy, 6 idiopathic, 3 Hu. **: 4 CANVAS/RFC1‐neuropathy, 3 idiopathic, 1 vitamin B12 deficiency, 1 cisplatin. ***: 1 idiopathic.

A total of 164 patients with SNN, CIDP, or LDN were included, and SRAR was analyzed in 90/164 (54.9%) of patients; 30/59 (50.8%) of patients with SNN, 30/43 (69.8%) of patients with CIDP, and 30/62 (54.9%) of patients with LDN (Figure 1). Among the 59 patients with SNN, 20 had a CANVAS/*RFC1*‐neuropathy, and SRAR was analyzed in only 6/20 (30%). A SRAR calculation could not be performed in 74/164 (45.1%) of the overall population, including 42/102 (41.2%) patients with NLDN, due to absent radial sensory or sural SNAPs.

Among patients for whom SRAR was analyzed, in patients with SNN, the mean age was 56.2 years (SD 13.9) and the male‐to‐female ratio was 1:1; in patients with CIDP, the mean age was 58.9 years (SD 13.14) and the male‐to‐female ratio was 2.3; and in patients with LDN, it was 64 years (SD 15.9) and the male‐to‐female ratio was 1.3. Etiologies of neuropathies for these 90 patients are detailed in Table 1.

**TABLE 1:** Etiology of neuropathy.

	SNN (*n* = 30)	CIDP (*n* = 30)	LDN (*n* = 30)
Immune mediated	9	—	5
Anti‐FGFR3	4	—	—
Sjögren	3	—	1
Anti‐Ago	2	—	—
Other CTD	—	—	4^b^
Inherited	7	—	1
CANVAS/*RFC1*	6	—	—
Friedreich	1	—	—
*TTR* amyloidosis	—	—	1
Vitamin B12 deficiency	1	—	2
Toxic	2	—	5
Cisplatin	2	—	—
Other chemotherapy	—	—	2
Nitrous oxide	—	—	2
Isoniazid	—	—	1
Diabetes mellitus	—	—	3
Paraneoplastic (Hu/CV2)	1	—	—
Critical illness neuropathy	—	—	1
Idiopathic	10^a^	—	13
Typical CIDP	—	19	—
Sensory‐predominant CIDP	—	7	—
Multifocal CIDP	—	4	—

Abbreviations: Ab, antibody; Anti‐Ago, Argonaute antibodies; CIDP, chronic inflammatory demyelinating polyradiculoneuropathy; CTD, connective tissue disease; LDN, length‐dependent neuropathies; SNN, sensory neuronopathy; TTR, transthyretin.

^a^ Three possibly inflammatory due to an immune context or non‐specific antibodies; four with context of alcohol abuse and/or malnutrition, and/or vitamin deficiency [25]; three without specific context.

^b^ Two with rheumatoid arthritis; one with rheumatoid arthritis and Sjögren; one with systemic lupus erythematosus and systemic sclerosis.

### 3.2 EDX Analyses and SRAR Thresholds Value

Table 2 summarizes the EDX characteristics of the 90 patients in whom the SRAR was analyzed. The median SRAR in the NLDN group was higher than that in the LDN group.

**TABLE 2:** Electrodiagnostic characteristics.

	NLDN (*n* = 60)			LDN (*n* = 30)
	SNN (*n* = 30)	CIDP (*n* = 30)	Total (*n* = 60)	
Sural SNAP amplitude, μV, median (IQR)	4.5 (3.0–6.0)	6.0 (4.0–11.0)	5.0 (3.8–7.3)	4.0 (3.0–5.8)
Radial SNAP amplitude, μV, median (IQR)	5.0 (4.0–9.0)	9.0 (5.3–18.8)	8.0 (5.0–12.3)	22.0 (18.5–33.8)
SRAR, median (IQR)	0.75 (0.55–1.00)	0.67 (0.42–1.24)	0.74 (0.50–1.00)	0.17 (0.12–0.23)
Low sural SNAP amplitude	30 (100.0%)	19 (63.3%)	49 (81.7%)	30 (100.0%)
Low radial SNAP amplitude	29 (96.7%)	22 (73.3%)	51 (85.0%)	7 (23.3%)
Length‐dependent EDX pattern^a^	18 (60.0%)	20 (66.7%)	38 (63.3%)	30 (100.0%)

Abbreviations: CIDP, chronic inflammatory demyelinating polyradiculoneuropathy; EDX, Electrodiagnostic studies; IQR, interquartile range; LDN, length‐dependent neuropathies; NLDN, non‐length‐dependent neuropathy; SNN, sensory neuronopathy; SRAR, sural radial amplitude ratio.

^a^ Length‐dependent EDX pattern: lower value of sural SNAP amplitude compared to radial SNAP amplitude [23].

Radial SNAP amplitudes were normal in 8/30 (26.7%) patients with CIDP and in one patient with SNN (3.3%), a 63‐year‐old male with cisplatin‐induced neuropathy, whose radial SNAP amplitude was marginally above the lower limit of normal, despite very low median and ulnar SNAP amplitudes.

**FIGURE 2:**
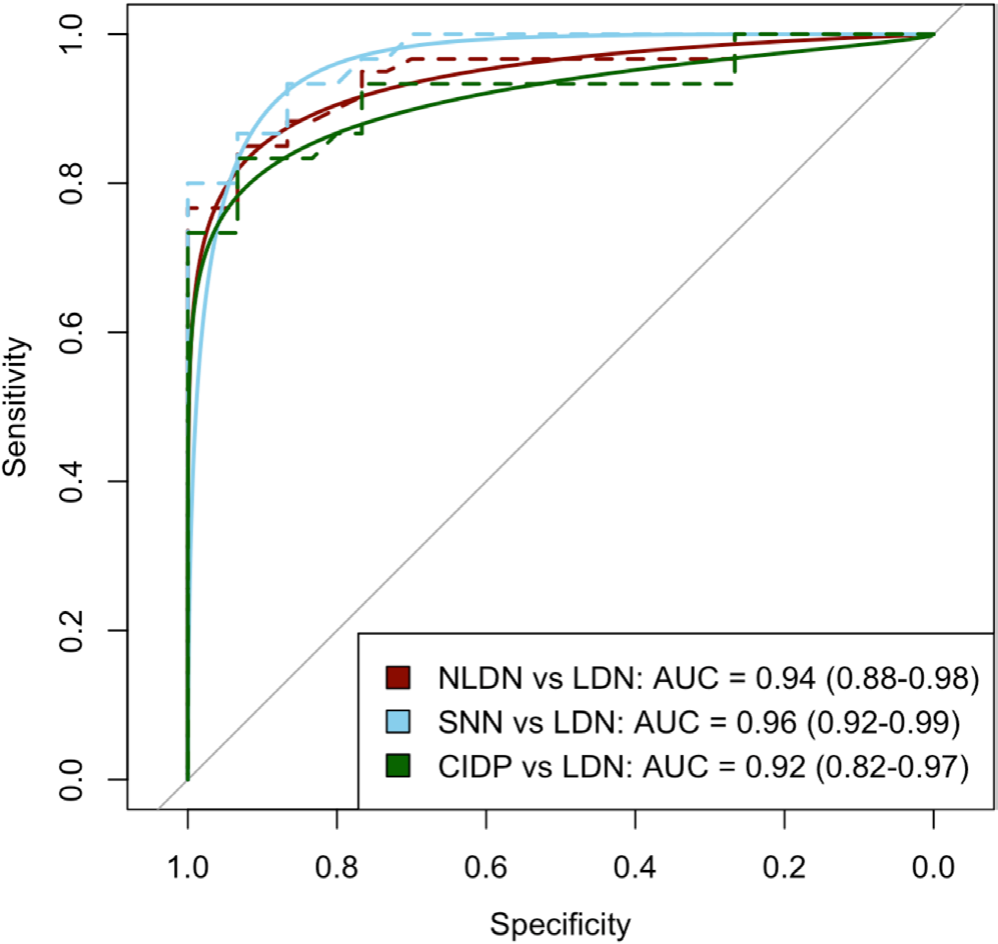
ROC curve of SRAR. CIDP, chronic inflammatory demyelinating polyradiculoneuropathy; LDN, length‐dependent neuropathies; NLDN, non‐length‐dependent neuropathy; SNN, sensory neuronopathy, SRAR, sural radial amplitude ratio. Different ROC curves were presented, all compared to LDN, in blue the SNN, in green the CIDP, and in red the NLDN group (so SNN and CIDP), which is why, the red curve had a value between the blue and the green curve.

Figure 2 presents the ROC curves for SRAR group analyses. The optimal calculated SRAR threshold to discriminate NLDN from LDN was 0.339 (95% CI: 0.29–0.40). To facilitate practical use, this threshold was rounded to 0.33 (equivalent to one‐third). Its diagnostic performance is detailed in Table 3. A sensitivity analysis assessed the optimal SRAR threshold using varying pre‐test probability and risk cut‐off preferences; the results are presented in Figure S1.

**TABLE 3:** The diagnostic performance of SRAR using the 0.33 threshold.

	SNN vs LDN	CIDP vs LDN	NLDN vs LDN
Sensitivity, % (CI)	91.7 (84.3–99.1)	80.6 (70.9–90.4)	84.4 (77.8–90.9)
Specificity, % (CI)	86.9 (79.7–94.0)	86.9 (79.7–94.0)	86.9 (79.7–94.0)
PPV, % (CI)	NA	NA	53.0 (38.3–67.7)
NPV, % (CI)	NA	NA	96.9 (95.7–98.2)

*Note*: The sensitivity, specificity, PPV, and NPV of the simple rounded optimal threshold (at 0.33) were calculated, assuming 15% pre‐test probability for non‐length‐dependent neuropathy (SNN or CIDP) for the PPV and NPV. The sensitivity and specificity of the SRAR to discriminate patients with SNN and CIDP from patients with length‐dependent neuropathy were assessed using the same rounded optimal threshold (at 0.33). Specificity was the same for all groups, due to a comparison with the same reference group of length‐dependent neuropathy. Specific pre‐test probability is unknown for SNN and CIDP, thus PPV and NPV were not calculated (NA).

Abbreviations: CI, confidence intervals; CIDP, chronic inflammatory demyelinating polyradiculoneuropathy; LDN, length‐dependent neuropathies; NA, not available; NPV, negative predictive value; PPV, positive predictive value; SNN, sensory neuronopathy; SRAR, sural radial nerve amplitude ratio.

SRAR was not significantly correlated with age (Pearson's correlation coefficient 0.01 [95% CI: −0.19, 0.22], *p* = 0.90) or sex (Wilcoxon, *p* = 0.62). Additionally, in patients with SNN, the SRAR was not significantly different among the three etiological categories (*p* = 0.15).

A length‐dependent EDX pattern was observed in 30/30 (100%) patients with LDN and 38/60 (63.3%) patients with NLDN (20/30 CIDP and 18/30 SNN). Among these 38 patients, the SRAR was greater than 0.33 in 30 (78.9%), including 15/20 (75%) patients with CIDP and 15/18 (83.3%) with SNN. In addition, among the 20 patients with CANVAS/*RFC1*‐neuropathy, the length‐dependent EDX pattern was found in 8/20 (40%); in the 6 patients for whom SRAR was analyzed, the values were greater than 0.33.

## 4 Discussion

This study found that a SRAR greater than 0.33 could discriminate NLDN from LDN with a sensitivity and specificity close to 85%.

SRAR may be useful in SNN, particularly in CANVAS/*RFC1*‐neuropathy [15]. In these patients, EDX can demonstrate a misleading length‐dependent pattern, which was found in 40% of our patients as well as in other cohorts [23, 26]. Ataxia can be absent in the early stages and most patients have symmetrical sensory involvement, frequently limited to the lower limbs; consequently, the Camdessanche criteria may not be met. Of note, these criteria were developed and then validated in a cohort in which only 5% of patients had inherited neuropathy, and none had CANVAS/*RFC1*‐neuropathy [3, 6, 15, 23, 26]. In all patients with CANVAS/*RFC1*‐neuropathy in whom a SRAR could be calculated in the current series, SRAR was > 0.33, suggesting its complementary role to the Camdessanche criteria. Secondly, in the early stages of an acquired SNN, SNAP amplitudes may remain detectable albeit reduced, potentially leading to diagnostic delays, because such studies do not meet EDX criteria [6]. Prospective studies are needed to validate the SRAR utility in those with early‐stage SNN. To date, SRAR has rarely been used in the diagnosis of NLDN, except to suggest SNN in spinocerebellar ataxia (SCA) using a threshold of 0.3 [12, 13, 14]. This previous threshold aligns with the present threshold of 0.33; however, the methodology used to calculate the threshold was not detailed in the previous study [12].

Early diagnosis and treatment are crucial to prevent axonal damage and long‐term disability in patients with CIDP [27]. EDX is the cornerstone of CIDP diagnosis and now includes the sural sparing pattern requiring a normal sural SNAP amplitude [1], which is not the case in two‐thirds of patients presented in the current study. Additionally, in clinical practice, the frequent occurrence of an abnormal median SNAP due to compressive neuropathy has led to the use of the radial sensory nerve herein [28, 29]. Given the two limitations inherent to the sural sparing pattern, and despite its value in the diagnosis of CIDP, the SRAR > 0.33 could be particularly valuable.

It was previously suggested that the SRAR may vary depending on age in healthy controls, with a trend toward a decrease when age increases [30, 31]. However, in the present study, SRAR variation was not correlated with age nor sex, possibly due to the sample size but especially to a greater influence of severity in patients with neuropathy compared to healthy individuals without neuropathy. Furthermore, the SRAR values were similar across different etiologies of sensory neuropathy, although only etiologies with a clear association with SNN were classified as immune‐mediated SNN; of note, immune disease such as rheumatoid arthritis, systemic lupus erythematosus, or systemic sclerosis were not. It still suggests that while a pathological SRAR was not specific to any etiology, it may be a marker of the non‐length‐dependent mechanism.

The first limitation of the present study was the inability to calculate SRAR in approximately half of the patients who consulted during the recruitment period, therefore limiting its applicability in all cases and requiring the consideration of additional criteria. Additionally, the SRAR was calculated using the lower values of the radial and sural nerves, compared to the usual use of higher values [8]. Notably, in several studies, healthy controls without signs or symptoms, and with normal SNAP amplitudes, can have a SRAR above our pathological threshold. Nevertheless, in clinical practice, SRAR should be employed only in patients exhibiting sensory signs and symptoms who have reduced SNAP amplitudes [9, 30, 31, 32]. The PPV found herein, greater than 50%, was moderate, reflecting the higher prevalence of LDN compared to CIDP and SNN. Moreover, using different values of and could yield different thresholds. The prevalence of NLDN is higher at our tertiary center compared to more general centers; a different threshold could be used, adapted to the prevalence (Supporting Information).

## 5 Conclusion

SRAR is an easy‐to‐use, quick, sensitive, and specific tool that could be useful in the electrophysiological work‐up of neuropathies, with a threshold > 0.33 suggesting NLDN. Its role as an adjunct to conventional criteria could be particularly useful in challenging cases such as early‐stage SNN, CANVAS/*RFC1*‐neuropathy, and atypical CIDP presentations. However, the calculation is feasible only in approximately half of patients. Prospective studies are needed to refine its application and explore its role in broader clinical settings.

## Author Contributions

**Antoine Pegat:** conceptualization, investigation, writing – original draft, methodology, validation, writing – review and editing, data curation. **Antoine Gavoille:** data curation, formal analysis, methodology, writing – original draft, writing – review and editing, validation. **Florent Cluse:** writing – review and editing, supervision, validation. **Martin Moussy:** validation, writing – review and editing, supervision. **Philippe Petiot:** validation, writing – review and editing, supervision. **Jean‐Philippe Camdessanché:** validation, writing – review and editing, supervision. **Françoise Bouhour:** writing – review and editing, supervision, validation.

## Ethics Statement

We confirm that we have read the Journal's position on issues involved in ethical publication and affirm that this report is consistent with those guidelines.

## Conflicts of Interest

The authors declare no conflicts of interest.

## Supporting information


**Figure S1:** Sensitivity analysis.

## Data Availability

The data that support the findings of this study are available from the corresponding author upon reasonable request.
