# Supplementary material for: Sural/Radial Amplitude Ratio: A Useful Tool to Diagnose Non‐Length‐Dependent Neuropathy
Source: Muscle Nerve. 2025 Oct 9;73(1):34–40. doi: 10.1002/mus.70046 (PMC12690015; doi:10.1002/mus.70046)
Supplement: Supplementary file 1 — Figure S1: Sensitivity analysis. [file MUS-73-34-s001.docx]

**Supplementary figure 1– Sensitivity analysis**


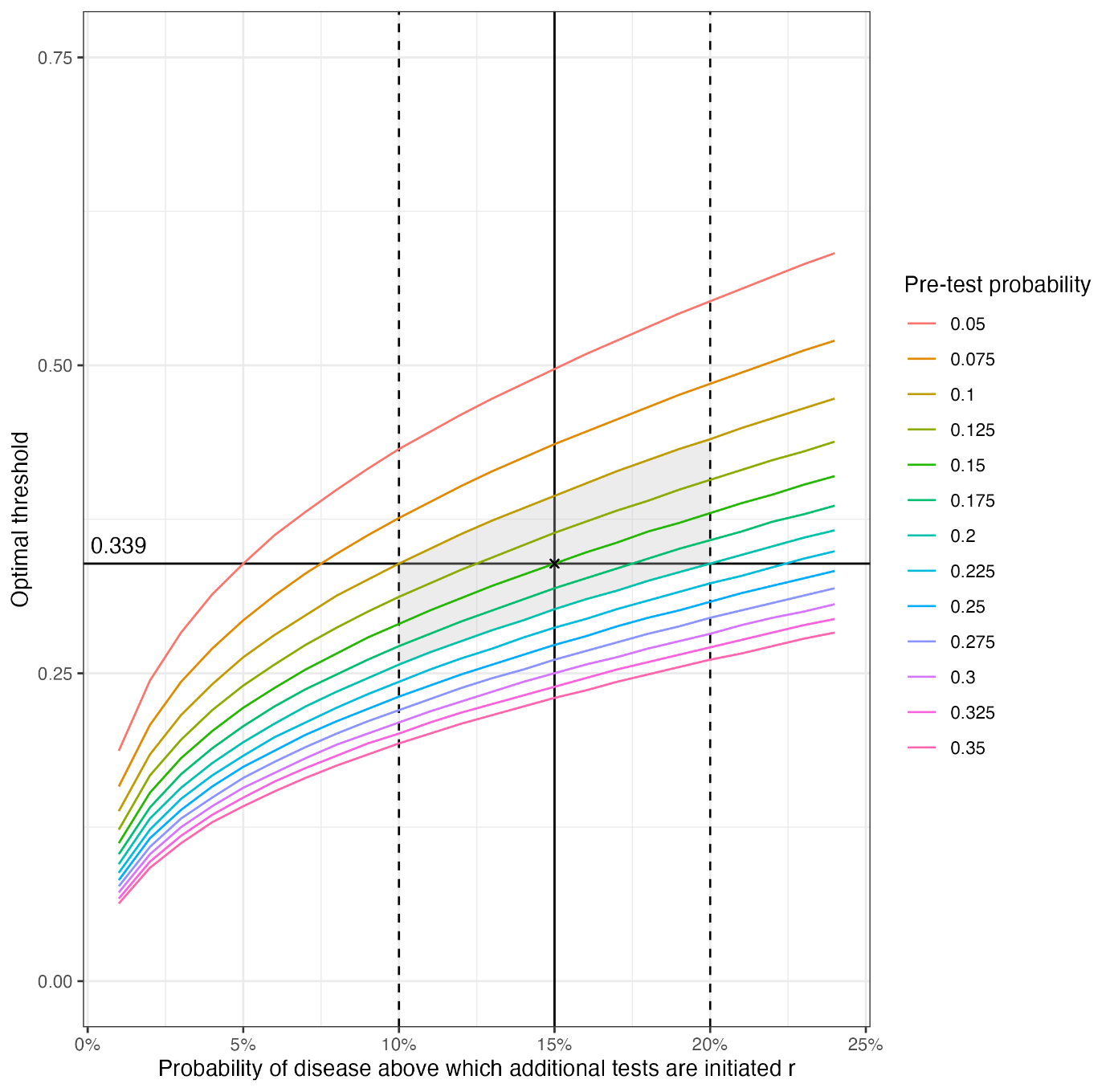


As a sensitivity analysis, the optimal threshold according to different hypotheses of pre-test probability and risk cut-off preference was performed. The calculated optimal threshold was 0.339 with our predefined pre-test probability at 15% (0.15), and the risk cut-off preference 𝑟 of 15%; after rounded at 0.33 to simplify the use in current practice.
